# Study of the impact of structural factors and cleaning efficiency in reducing hazardous litter density and its related pollution in urban environment

**DOI:** 10.1038/s41598-024-64645-w

**Published:** 2024-06-17

**Authors:** Maryam Morovati, Sheida Parsa, Maryam Panahande, Amin Hossaini Motlagh, Iman Parseh

**Affiliations:** 1 Department of Environmental Sciences and Engineering, Faculty of Agriculture and Natural Resources, Ardakan University, Ardakan, Iran; 2 Department of Environmental and Occupational Health, California State University, Northridge, USA; 3 Environmental Research Institute, Academic Center for Education, Culture and Research (ACECR), Rasht, Iran; 4 Department of Environmental Health Engineering, Faculty of Health, Yasuj University of Medical Sciences, Yasuj, Iran; 5 Department of Environmental Health Engineering, Behbahan Faculty of Medical Sciences, Behbahan, Iran

**Keywords:** Cigarette butt, PAHs, Urban cleaning system, Solid waste management, Environmental sciences, Environmental social sciences, Health care

## Abstract

Cigarette filter is the most common hazardous litter that contains many pollutants including PAHs. The durability of this litter in the urban environment has an important effect on the rate of pollutant leakage. In this study, the leakage rate of PAHs from the littered cigarette filters was estimated by considering the affecting parameters on their durability in the urban environment. The results showed that the density of littered cigarette filters in the studied locations was 0.00048–0.13563 g/m^2^. The maximum spatial variation of the littered cigarette filter was 225 times. The average leakage of the total studied PAHs was estimated to be 2.048 µg/10 m^2^. The impact of structural factors and efficiency of urban cleaning in the estimated leakage was at most 2.4 times. It is necessary to change the behavior of citizens in littering the cigarette filter, considering its durability in the urban environment, to reduce the environmental and health consequences caused by the leakage of PAHs.

## Introduction

One of the consequences of increasing urbanization and globalization in recent decades is the growth of urban solid waste generation^1,2^. The variety of available products and services due to industrial development and the increase in prosperity in the communities has also caused the composition of municipal solid waste to become more diverse^3^. In this situation, the emergence of new solid wastes with special characteristics is one of the challenges of municipal solid waste management^3^. Some types of emerging solid wastes in recent decades, including cigarette filters, are classified as hazardous wastes due to characteristics such as the leakage of chemical and toxic compounds^4,5^.

Seven decades ago, the filter was added to previous cigarettes with the aim of reducing the entry of cigarette smoke pollutants into the lungs of smokers, and today the most form of tobacco consumption is filtered cigars^6,7^. Although the filter has the ability to trap cigarette smoke pollutants and can be effective in reducing the harm to smokers, the generation of an emerging solid waste has been one of the consequences of filtered cigars^8^. Littering the cigarette filter as a solid waste caused by smoking is a common behavior by smokers^9,10^. This method of waste disposal has caused the cigarette filter to be reported as a widespread litter in public environments such as beaches and urban environments^11^. A study of different cities and beaches showed that cigarette filters have a significant contribution to the composition of litter. For example, studies carried out in Argentina, Germany, Spain, Brazil, and Iran reported that cigarette butts were the most frequent litter in the studied environments^12–15^. The small size and low visibility of cigarette filters in public and urban environments, especially on beaches, makes it very difficult to collect them^16^. In this situation, the durability of this litter in the environment will increase.

Increasing the durability of the cigarette filter in the environment will increase the leakage of trapped pollutants. Due to the presence of numerous chemicals in cigarette smoke, it is expected that the pollutants trapped in the cigarette filter very diverse^15^. For example, nicotine is one of the known toxin in the cigarette filter, which quickly leaks from it^14^. Also, heavy metals including chromium, cadmium, nickel, mercury, and lead have been detected in various amounts in cigarette filters, which have been proven to leak from it^15^. PAHs are important pollutants in cigarette filters that leak into the environment due to climatic conditions such as humidity^17^. In past studies, all types of PAHs have been detected in cigarette filters^17,18^. However, the leakage of PAHs from the cigarette filter to the environment depends on several factors, including the density of the littered cigarette filter, climatic conditions, durability of the littered cigarette filter in the environment, and the initial concentration of the pollutant. The behavior of smokers in disposing of cigarette filters, the structural characteristics of the environment, and the efficiency of the urban cleaning system have a direct impact on the mentioned parameters. The urban cleaning system in the studied city is similar to the pattern used throughout Iran. In this pattern, people called “pakban” collect litter manually using long brooms. Due to the possibility of resuspension of deposited particles on the surface and also to increase the speed of the process, this activity is carried out in the late hours of the night when there is minimal presence of citizens in the urban environment. Therefore, the aim of this study was to estimate the leakage of PAHs from the littered cigarette filter in the urban environment by considering the density of the littered cigarette filter, the structural characteristics of the environment, and the efficiency of the urban cleaning system.

## Method

**Figure 1:**
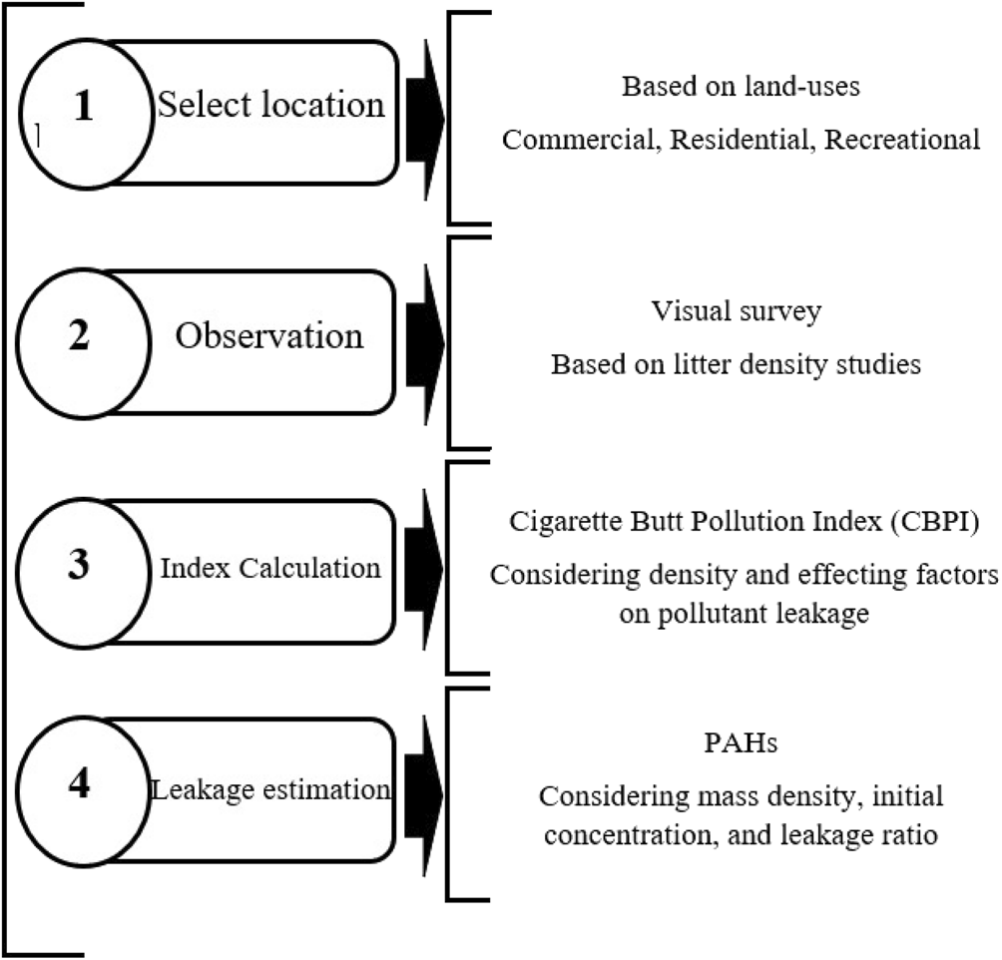
The order of the four stages of this study.

Data of the density of littered cigarette filters were collected by the method of visual survey of urban environments^16^. In this method, the defined surface including the width of the sidewalk plus one meter of the street width was visited and the number of littered cigarette filters was counted^16,19^. The evaluation of the density of littered cigarette filters in the studied locations was done in the evening hours and on working days^15,20^. To detect the average weight of littered cigarette filters, freshly smoked cigarette filters from ten popular cigarette brands in Iran were weighed^20^. As shown in Fig. 1, after collecting data of the density of littered cigarette filters, Cigarette Butt Pollution Index (CBPI) was used to interpret the status of the studied locations^20,21^. The details of the calculation of this index are shown in Table 1. Based on this, to calculate CBPI, applying E coefficient on the density of the littered cigarette filters was used.

**Table 1:** Formula, variables, and status classification of CBPI as used index in this study^20^.

Formula	CBPI = DCB × E										
Definitions	CBPI			DCB				E			
	Cigarette butt pollution index			Littered cigarette filter density (number/m^2^)				Coefficient			
E coefficient 10 ×	Distance to groundwater		Humidity			Structures				Land status	
	1 or 1.2 or 1.5 or 2		1 or 1.5 or 2 or 2.5			1 or 1.5 or 2				1 or 1.2 or 1.5 or 2.5	
Interpretation	< 1	1.1–2.5			2.6–5		5.1–7.5		7.6–10		> 10
	Very low pollution	Low pollution			Pollution		Significant pollution		High pollution		Sever pollution

The leakage of PAHs from the cigarette filter was estimated according to the initial concentration of these pollutants in the cigarette filter and its leakage potential in different climatic conditions^22^. The initial concentration of sixteen types of PAHs in cigarette filter has been reported by 0.48–5.76 µg/g^18^. Also, the rate of leakage of various types of PAHs from cigarette filters has been reported by 1–49%, which will increase under the influence of humidity^18^. Therefore, by identifying the density of littered cigarette filters in each location and the reported concentrations, the amount of PAHs leakage was estimated in each of the studied locations.

## Results and discussion

The results showed that there was no separate program for collecting littered cigarette butts in the studied city. Separate trash bins for cigarette butts were not installed in the urban environment. Littered cigarette butts were collected by the employees of the urban cleaning system (Pakbans) in the mass of littered wastes. In this system, the cleaning of low access points, such as tree pits and runoff collection channels, was often missed. The density of littered cigarette filters in the ten studied locations is shown in Table 2. The results showed that the density of cigarette filters was not the same in the studied locations and temporal and spatial variation were observed. The results showed that the minimum and maximum density of the littered cigarette filter were 0.00048 and 0.13563 g/m^2^, respectively. Spatial variation in the density of littered cigarette filters were evident. The average density in recreational land-use, residential land-use, and commercial land-use was 0.00051, 0.02349, and 0.11411 g/m^2^, respectively. The difference observed in the density of littered cigarette filters is directly related to the land-use. The effect of land-use on population density causes the possibility of cigarette filter littering in commercial land-use more than in residential and recreational land-uses^23^. However, in some places, the density of littered cigarette filters may be higher than the local average^24^. For example, in residential land-use, the number of littered cigarette filters is higher in places such as around supermarkets^16^. Also, more littered cigarette filters have been reported in recreational land-uses such as parks around benches^25^. These are points with high potential for cigarette filter littering and have an important effect on average density. Also, the phenomena affecting the population in the urban environment cause a change in the number and composition of litter, including cigarette filters. For example, one consequence of the Covid-19 pandemic was the reduction of people in the urban environment and the reduction of the possibility of waste littering^26,27^. In this situation, the difference in the number of littered cigarette filters in different land-uses will change.

**Table 2:** Density of littered cigarette filter in studied locations (g/m^2^).

	Categories									
Land-uses	Commercial				Residential				Recreational	
Locations	L1	L2	L3	L4	L5	L6	L7	L8	L9	L10
Density	0.13563	0.10851	0.08745	0.12486	0.02436	0.01959	0.02757	0.02247	0.00054	0.00048

**Figure 2:**
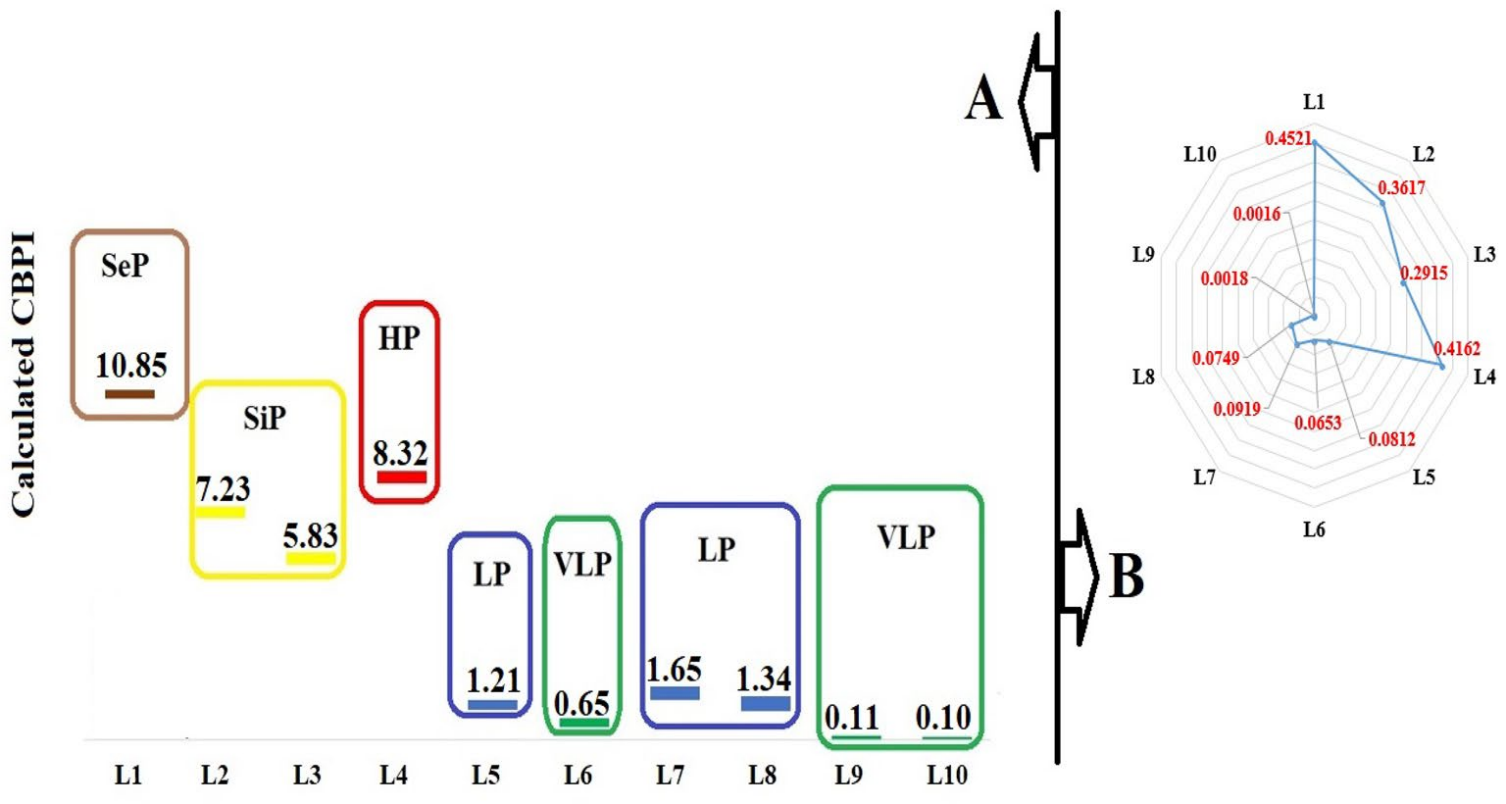
Comparison of pollution status in the studied locations. A, Based on CBPI (SeP, sever pollution; SiP, significant pollution; HP, high pollution; LP, low pollution; VLP, very low pollution); B, pollution density (filter/m^2^).

The density of littered cigarette filters has a direct effect on the leakage of trapped pollutants into the environment^22^. However, the effect of climatic conditions and the durability of cigarette filters are also effective in the leakage of trapped pollutants^9^. This effect is shown by comparing the density of cigarette filters with CBPI. As shown in Fig. 2, the density changes in the studied locations were different from the CBPI changes. The results showed that the lowest and highest CBPI were 0.1 and 10.850, respectively. Based on this, 30% of the studied locations were in significant pollution and worse status. The average CBPI in commercial land-use, residential land-use, and recreational land-use was 8.05, 1.21, and 0.106, respectively. The reason for the difference in CBPI is the difference in environmental conditions, which will cause a change in the E coefficient^28^. For example, the presence of three pits and also the surface runoff collection channels will increase the E coefficient^20^. The reason for this effect is the decrease of the efficiency of the urban cleaning system in these points, which are known as “low access points”^29^. Therefore, the increase of these points, which leads to an increase in the durability of littered cigarette filters due to the reduction of the efficiency of the cleaning system, is effective in increasing the leakage of trapped pollutants into the environment^20^. In addition, climatic conditions such as increasing humidity are effective in changing the E coefficient and increasing CBPI. Although the climatic conditions were the same in the studied area, however, the increase in humidity in recreational land-uses such as parks due to daily irrigation caused E coefficient increase compared to residential and commercial land-uses^20^.

Therefore, CBPI is a better index compared to the density of littered cigarette filters due to considering the factors affecting pollutant leakage into the environment^28^. The results of the leakage of various PAHs into the environment in the studied locations are shown in Table 3. The average leakage of these pollutants in the studied area was estimated at 0.204 µg/m^2^. The quantity of leakage of different types of PAHs into the environment was not the same, so that the highest and lowest amount of leakage were related to Naphthalene (0.1557 µg/m^2^) and Chrysene (0.00049 µg/m^2^), respectively. Also, due to the two effective factors in pollutant leakage into the environment, including density and structural-environmental factors, the variation in the amount of leakage in the studied land-uses was expected^15^. The results showed that the average leakage of total PAHs in commercial land-use, residential land-use, and recreational land-use was 0.426 µg/m^2^, 0.085 µg/m^2^, and 0.016 mg/m^2^, respectively. Based on this, littered cigarette filters are a source of PAHs leakage into the urban environment, which transfer different amounts of pollutants according to the initial concentration in the filter, the density of the filters, and the environmental conditions.

**Table 3:** Estimated PAHs leakage from littered cigarette filter in studied locations (µm/10 m^2^); IC, initial concentration; L, leaked concentration.

			PAH types															
			Naphthalene	Acenaphthylene	Acenaphthene	Fluorene	Anthracene	Phenanthrene	Fluoranthene	Pyrene	Benz_a_anthracene	Chrysene	Benzo_b_fluoranthene	Benzo_k_fluoranthene	Benzo_a_pyrene	indeno_123cd_pyrene	Benzo_ghi_perylene	Dibenz_a_h_anthracene
Locations	L1	IC	7.77	1.49	2.24	2.11	1.57	0.64	1.52	1.36	2.03	1.66	1.76	1.28	1.72	2.47	1	2.47
		L	3.85	0.14	0.47	0.16	0.01	0.02	0.03	0.03	0.02	0.01	0.02	0.01	0.02	0.03	0.03	0.24
	L2	IC	6.22	1.19	1.79	1.69	1.26	0.51	1.22	1.09	1.63	1.38	1.41	1.02	1.38	1.97	0.79	1.97
		L	3.08	0.11	0.37	0.12	0.01	0.02	0.03	0.02	0.01	0.01	0.01	0.01	0.01	0.02	0.02	0.19
	L3	IC	5.01	0.96	1.44	1.36	1.01	0.41	0.98	0.87	1.31	1.07	1.13	0.82	1.11	1.59	0.64	1.59
		L	2.48	0.09	0.30	0.10	0.01	0.01	0.02	0.01	0.01	0.01	0.01	0.01	0.01	0.02	0.02	0.15
	L4	IC	7.14	1.37	2.05	1.94	1.45	0.59	1.40	1.25	1.87	1.52	1.62	1.17	1.58	2.26	0.91	2.26
		L	3.54	0.13	0.43	0.14	0.01	0.02	0.03	0.02	0.02	0.01	0.02	0.01	0.01	0.02	0.02	0.22
	L5	IC	1.38	0.26	0.39	0.37	0.28	0.11	0.27	0.24	0.36	0.29	0.31	0.22	0.30	0.43	0.17	0.43
		L	0.68	0.02	0.08	0.03	0.003	0.004	0.007	0.005	0.003	0.002	0.003	0.002	0.003	0.004	0.004	0.042
	L6	IC	1.09	0.21	0.31	0.29	0.22	0.09	0.21	0.19	0.28	0.23	0.24	0.18	0.24	0.34	0.14	0.34
		L	0.54	0.02	0.06	0.02	0.002	0.003	0.005	0.003	0.002	0.002	0.002	0.002	0.002	0.004	0.003	0.033
	L7	IC	1.55	0.29	0.44	0.42	0.31	0.12	0.30	0.27	0.40	0.33	0.35	0.25	0.34	0.49	0.19	0.49
		L	0.76	0.03	0.09	0.03	0.003	0.005	0.007	0.005	0.004	0.003	0.003	0.003	0.003	0.005	0.005	0.048
	L8	IC	1.26	0.24	0.36	0.34	0.25	0.10	0.24	0.22	0.33	0.27	0.28	0.20	0.28	0.40	0.16	0.40
		L	0.62	0.02	0.075	0.025	0.003	0.004	0.006	0.004	0.003	0.002	0.003	0.002	0.003	0.004	0.004	0.039
	L9	IC	0.03	0.005	0.008	0.008	0.006	0.002	0.006	0.005	0.008	0.006	0.007	0.005	0.006	0.009	0.004	0.001
		L	0.014	0.0005	0.0016	0.0006	0.00007	0.00008	0.0001	0.0001	0.00008	0.00006	0.00007	0.00005	0.00006	0.0001	0.0001	0.0001
	L10	IC	0.02	0.005	0.007	0.007	0.005	0.002	0.005	0.004	0.007	0.005	0.006	0.004	0.006	0.008	0.003	0.008
		L	0.01	0.0005	0.0014	0.0005	0.00006	0.00008	0.0001	0.00008	0.00007	0.00005	0.00006	0.00004	0.00006	0.0009	0.00009	0.0007

Although the importance of this hazardous waste may be ignored due to its small size and the small amount of leakage per littered cigarette filter, however, considering the annual generation of eight trillion cigarette filters, its importance will be more understood^30^. Therefore, the abundance and dispersion as two characteristics of the cigarette filter make it a special source of leakage of various pollutants into the environment, including PAHs. A serious concern about pollutant leakage from littered cigarette filters is the contamination of soil and water resources. In previous studies, it has been proved that various types of pollutants, including nicotine, can leak from cigarette filters into water resources^14^. Also, in case of correct disposal of this waste by smokers and finally its landfill with municipal solid waste, there is a possibility of increasing landfill leachate pollutants, which has been analyzed and reported in the case of heavy metals^31^. Therefore, the health and environmental consequences caused by the leakage of trapped pollutants in cigarette filters are one of the reasons for paying attention to the management of this hazardous waste. For example, the consequences of exposure to PAHs include weakened immune systems, birth defects, genetic damage, damage to the nervous system, disruption of reproductive and hormonal systems, and cancer^32^.

The results showed that in addition to creating unpleasant landscapes in the urban environment, littered cigarette filters were an environmental threat due to the leakage of PAHs. Therefore, it is necessary to reduce the littering of cigarette filters by smokers due to the presence of low access points in urban environments and the low efficiency of the urban cleaning system in collecting littered cigarette filters^33^. Increasing the awareness of citizens and smokers about the environmental and health consequences related to littered cigarette filters can be effective in correcting their attitudes and changing behavior leading to a reduction in littering cigarette filters^10^. This approach is an effective act in “*reduction step*” of littered cigarette filters in the environment^16^. In addition, the reduction of littered cigarette filters will be provided by improving waste management structures, including the installation of special trash bins for the disposal of cigarette filters^30^. Considering the high proportion of cigarette filter littering even in cases where trash bins are installed in sufficient quantity and with short distance in the urban environment^14^, increasing the efficiency of the urban cleaning system as the most important approach in the “*removal step*” is necessary. For this purpose, it can be useful to use cleaning equipment adapted to urban structural characteristics and increase access, especially to low access points.

## Conclusion

The effect of the presence of low access points and the efficiency of the urban cleaning system on the density of littered cigarette filters and the leakage of PAHs into the urban environment was studied. The results showed that the average density of littered cigarette filters in commercial, residential, and recreational land-uses was 0.11411, 0.02349, and 0.00051 mg/m^2^, respectively. The cigarette butt pollution index in the studied locations was 0.1–10.850, which showed that 30% of the studied locations were in a very low pollution status and 50% of the studied locations were in a significant pollution and worse status. The average leakage of PAHs from the cigarette filter to the urban environment was estimated to be 0.2048 µg/m^2^. Accordingly, littered cigarette filters are a numerous and disperse source of PAHs in urban environments. In order to prevent health and environmental consequences caused by PAHs and other pollutants leaked from littered cigarette filters, it is necessary to increase the awareness of smokers and improve their behavior to reducing the proportion of cigarette filter littering in the urban environment. Also, the use of urban cleaning equipment with higher efficiency, especially in increasing the efficiency of cigarette filter collection in low access points, can reduce the durability of cigarette filters in the environment and reduce the uncontrolled leakage of pollutants.

## Data Availability

The datasets generated and analyzed during the current study were available from the corresponding author on reasonable request.
